# Arginine Regulates TOR Signaling Pathway through SLC38A9 in Abalone *Haliotis discus hannai*

**DOI:** 10.3390/cells10102552

**Published:** 2021-09-27

**Authors:** Yue Liu, Haixia Yu, Yanlin Guo, Dong Huang, Jiahuan Liu, Mingzhu Pan, Liu Wang, Wenbing Zhang, Kangsen Mai

**Affiliations:** 1 The Key Laboratory of Mariculture, Ministry of Education, Fisheries College, Ocean University of China, 5 Yushan Road, Qingdao 266003, China; liuyue@stu.ouc.edu.cn (Y.L.); yuhaixia@stu.ouc.edu.cn (H.Y.); guoyanlin@stu.ouc.edu.cn (Y.G.); huangdong@stu.ouc.edu.cn (D.H.); liujiahuan@stu.ouc.edu.cn (J.L.); pmz@stu.ouc.edu.cn (M.P.); wangliu@stu.ouc.edu.cn (L.W.); kmai@ouc.edu.cn (K.M.); 2 The Key Laboratory of Aquaculture Nutrition and Feeds, Ministry of Agriculture and Rural Affairs, Fisheries College, Ocean University of China, 5 Yushan Road, Qingdao 266003, China

**Keywords:** abalone, arginine, TOR, signaling pathway, SLC38A9

## Abstract

Arginine plays an important role in the regulation of the target of the rapamycin (TOR) signaling pathway, and Solute Carrier Family 38 Member 9 (SLC38A9) was identified to participate in the amino acid-dependent activation of TOR in humans. However, the regulations of arginine on the TOR signaling pathway in abalone are still unclear. In this study, *slc38a9* of abalone was cloned, and the *slc38a9* was knocked down and overexpressed to explore its function in the regulation of the TOR signaling pathway. The results showed that knockdown of *slc38a9* decreased the expression of *tor,* ribosomal s6 protein kinase (*s6k*) and eukaryotic translation initiation factor 4e (*eif4e*) and inhibited the activation of the TOR signaling pathway by arginine. Overexpression of *slc38a9* up-regulated the expression of TOR-related genes. In addition, hemocytes of abalone were treated with 0, 0.2, 0.5, 1, 2 and 4 mmol/L of arginine, and abalones were fed diets with 1.17%, 1.68% and 3.43% of arginine, respectively, for 120 days. Supplementation of arginine (0.5–4 mmol/L) increased the expressions of *slc38a9, tor, s6k* and *eif4e* in hemocytes, and abalone fed with 1.68% of dietary arginine showed higher mRNA levels of *slc38a9, tor, s6k* and *eif4e* and phosphorylation levels of TOR, S6 and 4E-BP. In conclusion, the TOR signaling pathway of abalone can be regulated by arginine, and SLC38A9 plays an essential role in this regulation.

## 1. Introduction

Arginine (Arg) is an essential amino acid for fish and neonatal mammals [1,2]. It is not only used as raw materials for protein synthesis but also as signal molecules to effectively regulate the synthesis and degradation of proteins [3,4]. Arginine is also involved in the secretion of several hormones, such as stimulating the somatotropic axis manifested by the secretion of growth hormone and insulin-like growth factor I [5,6]. In addition, it promotes the secretion of insulin, which could promote the absorption of glucose and amino acids [7,8]. In addition, arginine has several physiological functions, including the synthesis of urea, glutamic acid, creatine, proline, polyamines and nitric oxide (NO), inflammation and innate immune responses [1,9].

Protein synthesis is the foundation and key process of animal growth and development, which is limited by translation initiation [10,11]. The target of the rapamycin (TOR) signaling pathway is one of the most important pathways regulating protein metabolism by amino acids [12]. TOR regulates protein synthesis and initiates translation through stimulating ribosomal s6 protein kinase (S6K) activity and simultaneously inhibiting the binding of the eukaryotic translation initiation factor 4E-binding protein (4E-BP) to eukaryotic translation initiation factor 4E (eIF4E) [13,14,15]. In mammals, arginine regulates the mTOR signaling pathway at the transcriptional and protein levels both in vivo and in vitro [16,17,18,19,20]. In fish, arginine has been shown to regulate the expression of TOR-related genes and promote protein synthesis in grass carp (*Ctenopharyngodon idellus*) [21], hybrid grouper (*Epinephelus fuscoguttatus*♀ × Epinephelus lanceolatus♂) [22], blunt snout bream (*Megalobrama amblycephala*) [11], gibel carp (*Carassis auratus gibelio*) [23] and Jian carp (*Cyprinus carpio* var. Jian) [24]. However, no significant difference was observed in the expression of *tor, s6k* and *4e-bp* in tiger puffer (*Takifugu rubripes*) fed with different dietary arginine levels [25].

Amino acids serve as key stimuli of the TOR signaling pathway, but the mechanism of amino acids regulating the TOR signaling pathway was not fully understood. In mammals, Rag GTPases mediate the amino acid induced re-localization of TOR to the lysosomal surface, and Ragulator is responsible for tethering Rag GTPases to the lysosome and interacts with them in an amino acid- and v-ATPase-dependent manner [26,27]. In recent years, solute carrier family 38 member 9 (SLC38A9) was identified as a novel component of the Rag-Ragulator machinery, which plays an important role in sensing and transmitting amino acid signals to the TOR signaling pathway [28,29]. In HEK-293T cells, knockdown of SLC38A9 inhibited the activation of TOR by arginine, and overexpression of SLC38A9 made TOR signaling insensitive to amino acid starvation in human and mouse cells [28,30]. These studies indicated that SLC38A9 plays a crucial role in the activation of the TOR signaling pathway by amino acids, particularly arginine, but evidence is lacking in aquatic animals.

Abalone (*Haliotis discus hannai*) is large marine mollusks of archeogastropod [31]. Previous studies showed that the mRNA levels of TOR-related genes were regulated by dietary protein or lipid levels [32,33]. However, the regulation of amino acids on the TOR signaling pathway of abalone is still unclear. In the present study, the *slc38a9* of abalone was knocked down and overexpressed to better understand the molecular function and mechanism of SLC38A9 in the regulation of the TOR signaling pathway. The effect of arginine on the TOR signaling pathway was explored in vivo and in vitro. These multi-level characterizations provide a better mechanistic understanding of the regulation of the TOR signaling pathway by amino acids in marine invertebrates and provide evidence for the traces of evolution of TOR.

## 2. Materials and Methods

### 2.1. Ethical Statement

This study involved in vivo and in vitro experiments, and all care and handling of abalones were approved by the Animal Care and Use Committee of Ocean University of China (Approval No. OUC-SMP-2020-0126, January, 2020).

### 2.2. Molecular Cloning, Sequence Analysis and Tissue Distribution of slc38a9 in Abalone

#### 2.2.1. Experimental Animals and Sample Collection

Abalones (Weight: 20.0 ± 0.2 g) were obtained from an aquafarm in Weihai, Shandong province, China. They were temporarily cultured in a re-circulating water system with an ultraviolet sterilization lamp for 2 weeks. Before sampling, abalones were anesthetized with 5% of ethanol. The foot muscle was gashed with a scalpel to collect the hemolymph, and then muscle, mental, gill, intestine, gonad and digestive gland were isolated. All samples were frozen in liquid nitrogen and kept in −80 °C.

#### 2.2.2. Total RNA Extraction and Reverse Transcription

The RNA from the digestive gland was extracted using FastPure^®^Cell/Tissue Total RNA isolation Kit V2 (RC112-01 50 rxn, Vazyme, Nanjing, China). Other tissues were lysed with RNAiso Plus Kit (9109; Takara Biotech, Beijing, China) to extract the total RNA. The purity and concentration of the total RNA were quantified by Nanodrop 2000/2000C spectrophotometer (Thermo Fisher Scientific, Waltham, MA, USA) based on the ratio of A260/A280, and the integrity was tested by 1.5% denaturing agarose gel electrophoresis. Then RNA was treated with DNase and reverse transcribed into cDNA using the Prime ScriptTM RT reagent Kit (RR047A; Takara Biotech, Beijing, China).

#### 2.2.3. Molecular Cloning of *slc38a9*

The method of *slc38a9* cloning was referred to Wang et al. [34]. The mRNA sequence of *slc38a9* was obtained from the transcriptome database (unpublished data), and the primers were designed by Oligo 7 software (Molecular Biology Insights, Inc., Colorado Springs, CO) and synthesized by Sangon Biotech (Shanghai, China) (Table 1). cDNA of abalone muscle was used as a template for PCR, and the products were detected by 1% agarose gel electrophoresis. The target band was recovered using the SanPrep Column DNA Gel Extraction Kit (Sangon Biotech, Shanghai, China). The recovered product was linked to a blunt cloning vector and transferred to Trans1-T1 phage resistant chemically competent cells (TransGen Biotech, Beijing, China). The bacteria solution was culture expanded and applied evenly on LB solid medium containing ampicillin. Positive colonies were selected after overnight culture and verified using colony PCR, the correct colonies were selected and sequenced by Sangon Biotech (Shanghai, China).

#### 2.2.4. Sequence Analysis of *slc38a9*

Blast retrieval and comparison of the complete sequence were performed on National Center for Biotechnology Information (Available online: https://www.ncbi.nlm.nih.gov/orffinder/, accessed on 8 May 2020). The amino acid sequence of abalone *slc38a9* was deduced and analyzed by ExPASy expert protein analysis system (Available online: http://web.expasy.org/protparam/, accessed on 8 May 2020), and sequences of other species were downloaded from the NCBI database. Multiple alignment of these amino acid sequences was performed by DNAMAN 6.0 (Lynnon Biosoft, San Ramon, CA, USA). Phylogenetic analysis was carried out by the neighbor-joining method using MEGA X, and bootstrap values (%) of 1000 replicates were calculated for each node of the consensus tree obtained. SignalP 4.1 (Available online: http://www.cbs.dtu.dk/services/SignalP/, accessed on 8 October 2020) was used to detect the signal peptide, and transmembrane domains were predicted using the TMHMM Server v.2.0 (Available online: http://www.cbs.dtu.dk/services/TMHMM/, accessed on 21 October 2020). Furthermore, the secondary and three-dimensional structures of the sequence were predicted by PredictProtein (Available online:http://www.predictprotein.org/, accessed on 21 October 2020) and SWISS-MODEL (Available online: https://swissmodel.expasy.org/, accessed on 21 October 2020).

#### 2.2.5. Tissue Distribution of *slc38a9*

Six abalones were used for the tissue distribution analysis of *slc38a9*, the expression levels of *slc38a9* in seven tissues (hemolymph, muscle, mental, gill, intestine, gonad and digestive gland) of abalone were detected by quantitative real-time PCR.

### 2.3. Function Analysis of slc38a9

#### 2.3.1. Synthesis and Injection of *slc38a9* siRNA

The sequence of negative control siRNA (siRNA-NC) and synthesis of siRNA was conducted by Wang et al. [34]. Four siRNAs (siRNA-108, siRNA-236, siRNA-347 and siRNA-469) targeting different encoding regions of *slc38a9* were designed by BLOCK-iT™ RNAi Designer (Available online: http://rnaidesigner.thermofisher.com/rnaiexpress/, accessed on 20 May 2020) (Table 2). Primers of these siRNAs were synthesized by Sangon Biotech and siRNA duplexes were synthesized using the T7 RNAi Transcription Kit (TR102-01, Vazyme, Nanjing, China). The concentration of all the siRNAs was uniformly adjusted to 300 ng/μL.

Abalones (Weight: 19.8 ± 0.3 g) were acclimatized to the laboratory environment for two weeks before the experiment. To explore the efficiency of these siRNAs, six groups (6 abalones each group) of abalones were injected intramuscularly with 100 μL PBS, siRNA-NC, siRNA-108, siRNA-236, siRNA-347 or siRNA-469. Abalones injected with PBS served as the control group. The muscles of each the abalone were sampled 12 h after injection. Then 60 abalones were randomly divided into two groups and injected with 100 μL siRNA-NC or siRNA-347, respectively. The muscles were sampled at 0, 6, 12, 24 and 48 h after injection (6 abalones each time) to explore the efficiency of siRNA at different times.

#### 2.3.2. Overexpression Plasmid Construction and Injection

The overexpression plasmid was constructed according to the protocols described by Liu et al. [35]. The sequence of the *slc38a9* open reading frame (ORF) was obtained according to the step in Section 2.2.3. Homologous arms of Bam HI region in pcDNA3.1 were added to the *slc38a9* by the PCR of the templates of *slc38a9* with the forward primer *slc38a9*-HR-F (5′ cttggtaccgagctcggatccATGGGGAGAGGAAGTCGCA 3′) and the reverse primer *slc38a9*-HR-R (5′ ccacactggactagtggatccTGTTGTATGGCCGATGATGAGG 3′). The target gene sequence was linked to the pcDNA3.1 digested with the Bam H I enzyme, and then transferred to Trans-T competent cells. The extracted plasmid is sequenced to verify that the sequence is attached to the vector. The bacteria successfully linked to the target gene were expanded by shaking the flask culture overnight, and adequate plasmid was collected using the EasyPure HiPure Plasmid MaxiPrep Kit (TransGen Biotech, Beijing, China). The concentration of the plasmid was eventually diluted to 300 ng/μL.

Abalones (Weight: 19.8 ± 0.3 g) were randomly divided into three groups (6 abalones each group) and injected intramuscularly with 100 μL PBS, pcDNA3.1 or pcDNA3.1-*slc38a9*. Abalones injected with PBS served as the control group. The muscles of abalone were sampled at 12 h after injection.

#### 2.3.3. Oral Administration of Arginine after siRNA Injection

Two groups (12 abalones each group) of abalones (Weight: 19.8 ± 0.3 g) were injected with 100 μL siRNA-NC or siRNA-*slc38a9*, respectively. After 12 h, abalones of each group were subdivided into two groups and orally administered with 100 μL of arginine (0.6 M) (Sigma, St. Louis, MO, USA) or PBS, and abalones injected with siRNA-NC and fed with PBS served as the control group. Muscles were collected 3 h later and immediately frozen in liquid nitrogen, then stored frozen at −80 °C for the following analysis.

#### 2.3.4. siRNA Injection and Feeding

In regard to the feeding model, 200 abalones (weight: 19.8 ± 0.3 g) were randomly divided into two groups. For one group, abalones were fed with basal diet (1.23% arginine), and the other group was fed with the diet with arginine added (1.72% arginine). The diet formula is shown in Table 3, and abalones fed with the basal diet served as the control (Con) group. After a 2-week feeding, abalones of each group were divided into two sub-groups and injected with 100 μL of siRNA-NC or siRNA-*slc38a9*. After 12 h, abalones were fed with the original diet again. Muscles of abalone were sampled at 0, 2, 3, 6, 9, 12 and 24 h after feeding, and 6 abalones were sampled from each sub-group at each time point.

### 2.4. Arginine Treatment of Abalone In Vitro and In Vivo

#### 2.4.1. Primary Cells Culture and Arginine Treatment

Abalones (Weight: 20.0 ± 0.2 g) were temporarily cultured in the re-circulating water system with an ultraviolet sterilization lamp. After that, abalones were anesthetized with 5% of ethanol. The body surface was wiped and sterilized with 75% ethanol. Foot muscle was gashed with a sterile scalpel, and then the hemolymph was collected and placed into the anticoagulant tube immediately. The complete medium consisted of Leibovitz’s L-15 Medium (Thermo Fisher Scientific, Waltham, MA, USA), 15% fetal bovine serum (FBS) (Bioind, Kibbuiz, Israel), and four antibiotics (100 uL/mL penicillin-streptomycin, 250 μg/mL gentamicin and 2 μg/mL amphotericin) (Solarbio, Beijing, China). The hemolymph was mixed with the complete medium in a ratio of 1:3 and plated into 6-well plates (Corning, Lowell, MA, USA) and then incubated at 22 °C.

Before the experiment, cells were washed with phosphate buffer solution (PBS) (HyClone, Logan, UT, USA) for three times and then cultured in the DMEM medium without arginine (Thermo Fisher Scientific, Waltham, MA, USA). After 12 h, cells were washed with PBS for another time and cultured in the DMEM medium with antibiotics and different arginine concentrations (0, 0.2, 0.5, 1, 2 and 4 mmol/L), respectively, (three replicates per concentration). Cells were sampled 9 h later.

#### 2.4.2. Feeding Trial

Abalones (Weight: 15.7 ± 0.03 g) were obtained from a fishery company in Pingtan, Fujian, China. Three experimental diets with different levels of arginine (1.17%, 1.68% and 3.43%) were designed (Table 4). Abalones were randomly assigned into nine tanks (60 abalones per tank, and three tanks for one group) and fed with the experimental diets for 120 days. Abalones were fed every two days (15:00–17:00). During the 120-day feeding trial, the water temperature was 24.4 ± 4.3 °C, pH was 7.63 ± 0.74 and the dissolved oxygen was higher than 6.9 mg·L^−1^. After the feeding trial, abalones were fasted for 72 h before sampling. The muscles and digestive gland from each abalone were collected and immediately frozen in liquid nitrogen, then stored at −80 °C.

### 2.5. Sample Analysis

#### 2.5.1. Quantitative Real-Time PCR

The expressions of *slc38a9* and TOR-related genes of abalone were detected by qRT-PCR using the 2× ChamQ Universal SYBR qPCR Master Mix (Q711–02, Vazyme Biotech, Nanjing, China). The cDNA of abalone tissue was used as the template for qRT-PCR. All the primers were designed by Oligo 7 software according to the sequence from NCBI and synthesized by Sangon Biotech (Shanghai, China), and the primer sequences are listed in Table 1. The qRT-PCR condition was as follows: one cycle of 95 °C for 30 s, followed by 40 cycles of 95 °C for 10 s, 58 °C for 30 s, a cycle 95 °C for 15 s and 60 °C for 60 s. β-actin gene was used as the reference gene to normalize the mRNA expression levels. The Ct values were calculated by using 2^−ΔΔCt^ method to quantitate the relative expression levels of target genes.

#### 2.5.2. Western Blot Analysis

Abalone muscles (30–40 mg) were homogenized and lysed in RIPA (Solarbio Science and Technology Co., Ltd., Beijing, China) with protease inhibitor and phosphatase inhibitor (Thermo Fisher scientific, Waltham, MA, USA). The homogenates were set on ice for 10 min and then centrifuged at 4 °C to collect the supernatant. Protein concentrations were determined using the BCA protein assay kit (Beyotime Biotechnology, Shanghai, China) according to the manufacturer’s instructions, and then all the samples were adjusted to the same concentration. Equal amounts of protein were subjected to SDS-PAGE gel electrophoresis and then electrophoretically transferred to PDVF membrane. The PVDF membrane was blocked with 5% non-fat powdered milk for 1h and incubated with the antibody overnight at 4 °C. The primary antibodies used are as follows: phospho-mTOR (Ser2448) (2971), mTOR (2972), phospho-S6 (Ser235/236) (4858), S6 (2217), phospho-4E-BP1 (Thr37/46) (2855) and 4E-BP1 (9644) were purchased from Cell Signaling Technology Inc (Danvers, MA, USA). Then the PVDF membranes were washed 3 times and incubated with HRP-labeled Goat Anti-Rabbit/Mouse IgG (Beyotime Biotechnology, Shanghai, China) for 1 h at room temperature and enhanced chemiluminescence by Beyo ECL Plus reagents (Beyotime Biotechnology, Shanghai, China). The bands were quantified by densitometry using ImageJ software (Ver. 1.53, National Institutes of Health, Bethesda, MD, USA). GAPDH (AB-P-R001, Goodhere Biotechnology, Hangzhou, China) was the reference protein to normalize the target protein abundance and the phosphorylation level was calculated by the intensity ratio of phosphorylated protein to total protein.

### 2.6. Statistical Analysis

All data were analyzed by SPSS 25.0 software (IBM Corp., Armonk, NY, USA) and presented as mean ± SEM. Normality test and homogeneity of variances were performed before statistical analysis. T-test was used for the analysis of the two sets of data. Data with more than 2 sets were analyzed by one-way analysis of variance (ANOVA) followed by the Tukey’s multiple range test. Probabilities of *p* < 0.05 were considered statistically significant.

## 3. Results

### 3.1. Molecular Cloning, Sequence Analysis and Tissue Distribution of slc38a9 in Abalone

#### 3.1.1. Characterization and Phylogenetic Analysis of the *slc38a9*

**Figure 1:**
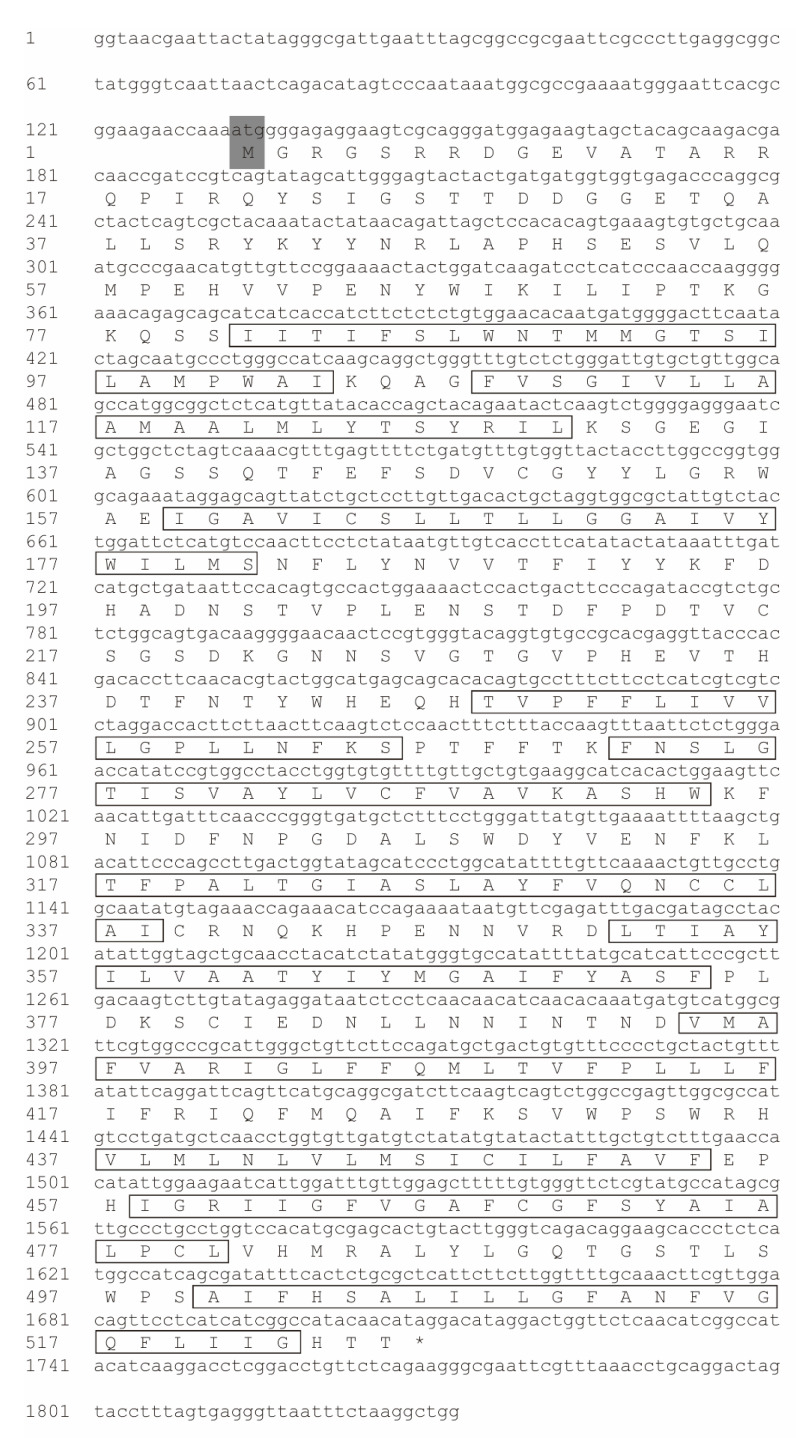
Nucleotide and deduced amino acid sequences of Solute Carrier Family 38 Member 9 (*slc38a9*). The initiation codon (ATG) is shaded in grey and the asterisks (*) indicate the translation stop codon (TAG). The predicted transmembrane regions are indicated in rectangular boxes.

**Figure 2:**
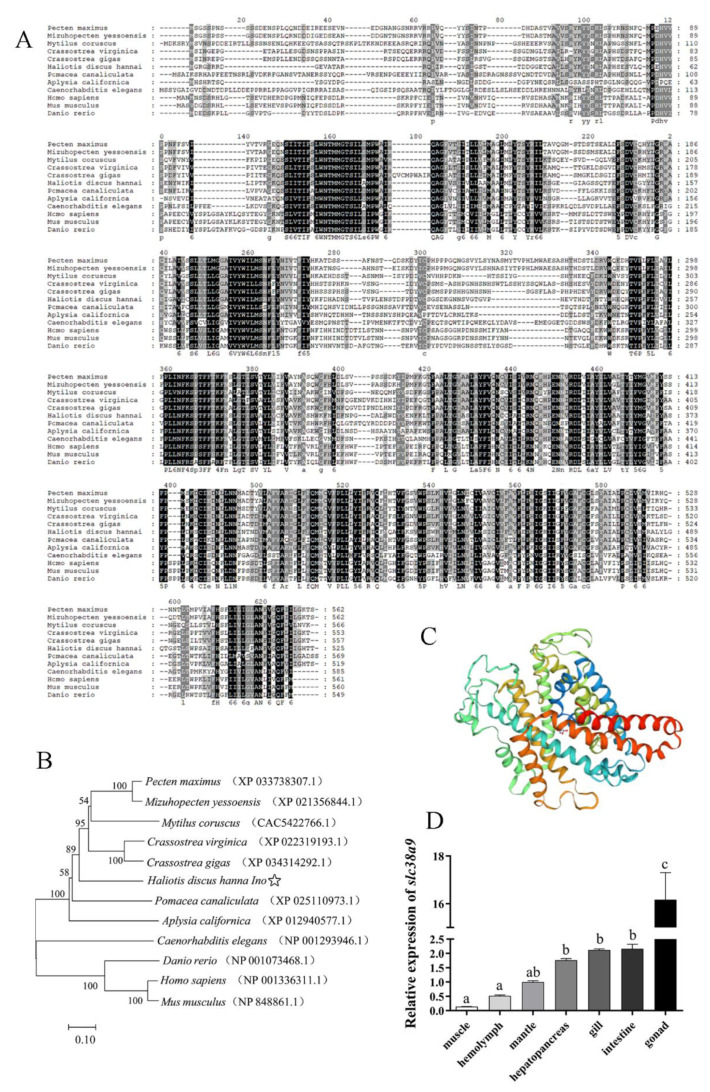
Sequence, structural, phylogenetic, and distribution analysis results of SLC38A9 in abalone. (**A**) Multiple alignment of amino acid sequence of *slc38a9*, identity amino acids are shown in black and homologous amino acids in grey. (**B**) The phylogenetic tree of *slc38a9* sequences, abalone is highlighted with “☆”. The numbers represent bootstrap percentages with the tree topology presented after 1000 replicates. The accession number was indicated after the species name. (**C**) Predicted tertiary structure of abalone SLC38A9 protein. (**D**) Tissues distribution of *slc38a9* in abalone. Results are represented as mean ± SEM (*n* = 3 replicate experiments), and different lowercase letters (a < b < c) indicate significant differences among tissues (*p* < 0.05).

The nucleotide sequence of cDNA and deduced amino acid sequence of *slc38a9* are shown in Figure 1. The sequence has been uploaded to NCBI (Accession No. MW390888). The full length of *slc38a9* sequence contains 1832 nucleotides, including an ORF of 1578bp. It codes a polypeptide of 525 amino acids with a molecular weight of 58.8 kDa and a theoretical isoelectric point (pI) of 7.20. The secondary structure was mainly alpha-helix, and the deduced protein includes 11 transmembrane domains. No signal peptide was found of the sequence. The predicted tertiary structure of abalone SLC38A9 protein is shown in Figure 2C.

Multiple alignment and phylogenetic tree for *slc38a9* and other counterparts are shown in Figure 2A,B. The results showed that *slc38a9* of abalone had significant homology with mollusk, including *Crassostrea gigas*, *Crassostrea virginica*, *Mytilus coruscus*, *Pecten maximus*, *Mizuhopecten yessoensis*, *Pomacea canaliculata*. Among these, *M. yessoensis* had the highest sequence consistency with abalone. All mollusk species formed a clade separate from zebrafish, mice and humans.

#### 3.1.2. Expression Analysis of *slc38a9* in Different Tissues of Abalone

Transcript levels of *slc38a9* in different tissues including muscle, hemocytes, mantle, gill, gonad, digestive gland and intestine were investigated to determine the tissue-specific expression profile of *slc38a9* in abalone. The result showed that *slc38a9* was widely expressed in all tested tissues, and it was most highly expressed in the gonad (*p* < 0.05), followed by intestine, gill, digestive gland and mantle. The lowest expressions were found in hemocytes and muscle (Figure 2D).

### 3.2. Function Analysis of slc38a9

#### 3.2.1. Expressions of TOR Pathway Related Genes after siRNA Injection

**Figure 3:**
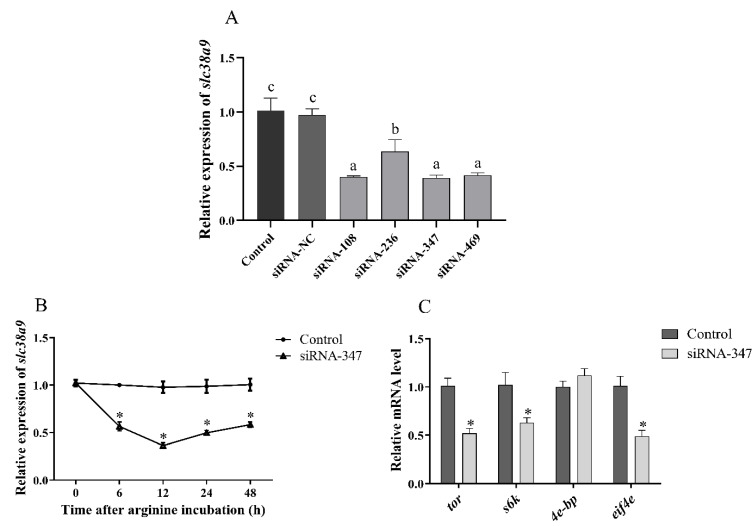
siRNA-*slc38a9* inhibits the expression of *slc38a9* and the TOR signaling pathway. (**A**) The relative expression levels of *slc38a9* in the muscle of abalones after injection with siRNA. (**B**) The relative expression levels of *slc38a9* in muscle at different time after injection with siRNA-347. (**C**) The relative expression levels of target of rapamycin (*tor*), ribosomal s6 protein kinase (*s6k*), eukaryotic translation initiation factor 4e binding protein (*4e-bp*) and eukaryotic translation initiation factor 4e (*eif4e*) in muscle at 12 h after *slc38a9* knockdown. Results are represented as mean ± SEM. Different lowercase letters (a < b < c) indicate significant differences among groups (*p* < 0.05), and asterisk (*) indicates a significant difference between the control and the experimental group (*p* < 0.05).

The silencing efficiency of four siRNA was measured to screen the most effective one. Compared with the control group, siRNA-NC had no significant effect on the mRNA level of *slc38a9* (*p* > 0.05), and the relative expression level of *slc38a9* was significantly decreased in all groups injected with siRNA-*slc38a9* (*p* < 0.05). The silencing efficiencies of siRNA-108, siRNA-347 and siRNA-469 were about 60% without significant difference among the three (*p* > 0.05), silencing efficiency of siRNA-236 was 40% (Figure 3A). To determine the time with the highest efficiency of siRNA, abalones were sampled at different time after injection. The result showed that the relative expression of *slc38a9* was significantly inhibited at 6 h after injection, the inhibition efficiency was highest at 12 h after injection, and the relative expression was still significantly inhibited at 48 h (*p* < 0.05) (Figure 3B).

Compared with the control group, the relative expression levels of *tor*, *s6k* and *eif4e* were significantly reduced after the injection of siRNA-*slc38a9* (*p* < 0.05), but there was no significant change in mRNA level of *4e-bp* (*p* > 0.05) (Figure 3C).

#### 3.2.2. Expressions of TOR Pathway Related Genes after Injection with pcDNA3.1-*slc38a9*

**Figure 4:**
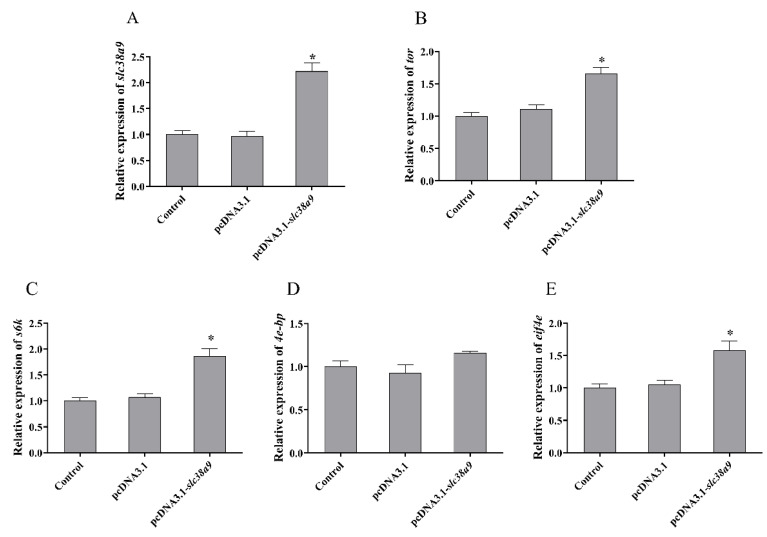
Effect of *slc38a9* overexpression on the TOR signaling pathway in abalone. The relative expression levels of (**A**) *slc38a9*, (**B**) *tor*, (**C**) *s6k*, (**D**) *4e-bp* and (**E**) *eif4e* in abalones after injection with plasmid. Results are represented as mean ± SEM (*n* = 3 replicate experiments), and asterisk (*) indicates a significant difference compared with the control group (*p* < 0.05).

Compared with the control group, intramuscular injection of pcDNA3.1 plasmid had no significant influence on the relative expression of *slc38a9* (*p* > 0.05), and the relative expression of *slc38a9* was up-regulated by pcDNA3.1-*slc38a9* plasmid injection significantly (*p* < 0.05). The mRNA levels of *tor*, *s6k* and *eif4e* were significantly increased after the intramuscular injection of pcDNA3.1-*slc38a9* plasmid (*p* < 0.05) (Figure 4).

#### 3.2.3. Expressions of TOR Pathway Related Genes after Injection of siRNA and Oral Administration of Arginine

**Figure 5:**
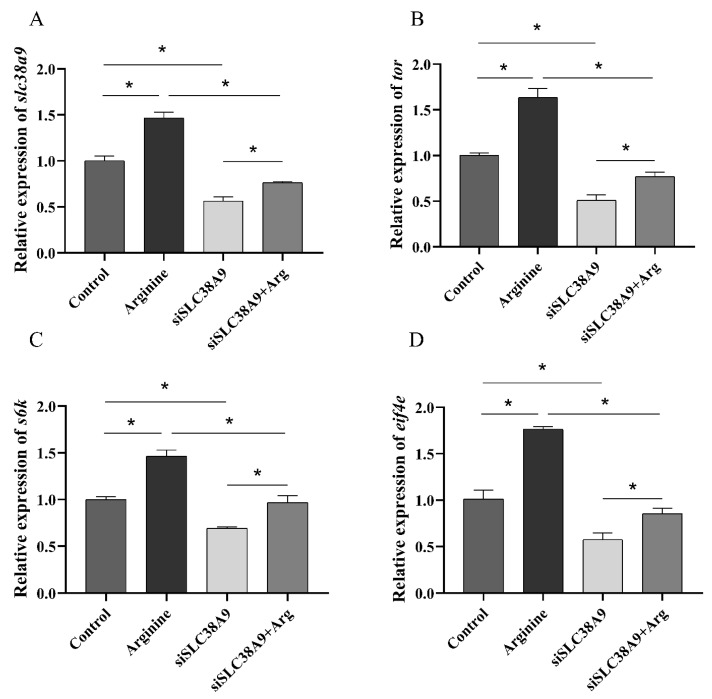
Effect of *slc38a9* knockdown on arginine-induced activation of the TOR signaling pathway in muscle. Abalones were orally administered arginine (0.1 mmol/L, 100 μL) or PBS after *slc38a9* knockdown. The figures show the expression levels of (**A**) *slc38a9*, (**B**) *tor*, (**C**) *s6k* and (**D**) *eif4e* in muscles after oral administration. Results are represented as mean ± SEM (*n* = 3 replicate experiments), and asterisk (*) indicate a significant difference between the two groups (*p* < 0.05).

Compared with the control group, oral administration of arginine significantly increased the expression levels of *slc38a9, tor*, *s6k* and *eif4e* in the muscles of abalone (*p* < 0.05). siRNA-mediated knockdown of *slc38a9* significantly inhibited the activation of the TOR signaling pathway by arginine and decreased the expression of *tor*, *s6k* and *eif4e* (*p* < 0.05) (Figure 5).

#### 3.2.4. Expressions of TOR Pathway Related Genes after Feeding and siRNA Injection

**Figure 6:**
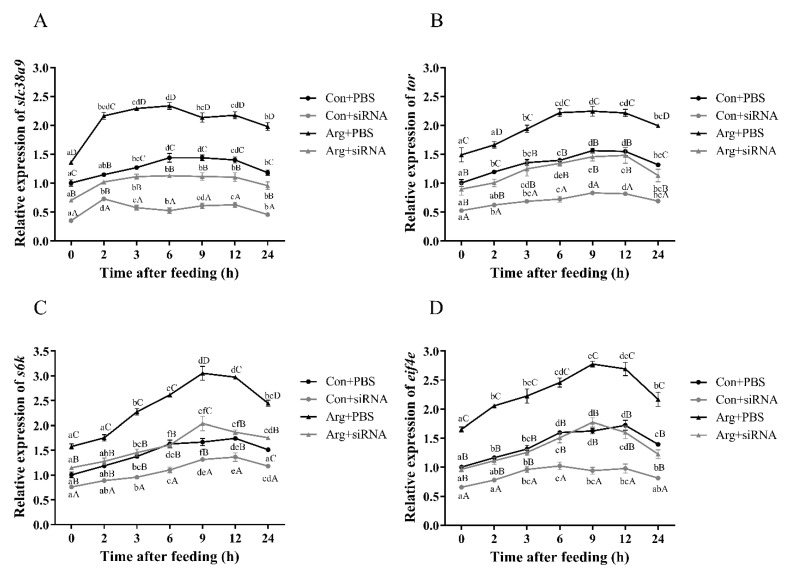
Effect of *slc38a9* knockdown on the activation of the TOR signaling pathway by dietary arginine. Abalones were fed with basal (1.23% arginine) and arginine-added (1.72% arginine) diets for two weeks before siRNA knockdown, abalones fed with basal diet served as the control group. After 12 h of siRNA injection, abalones were fed with the original diets. The figure shows the relative expression levels of (**A**) *slc38a9*, (**B**) *tor*, (**C**) *s6k* and (**D**) *eif4e* in muscle at different times after feeding. Results are represented as mean ± SEM, *n* = 3 replicate experiments. Different lowercase letters (a < b < c < d) indicate significant differences of the same group at different times, and capital letters (A < B < C < D) indicate the significance of different treatments at the same time (*p* < 0.05).

Compared with the basal diet, the arginine-added diet increased the transcript levels of *slc38a9*, *tor*, *s6k* and *eif4e* in abalone (*p* < 0.05). The expression of *slc38a9* had a peak after 6 h of feeding, and the expression levels of other genes reached the peak at 9 h (Figure 6). Furthermore, feeding after siRNA-*slc38a9* injection also up-regulated the expression levels of *slc38a9*, *tor*, *s6k* and *eif4e* (*p* < 0.05); however, the expression levels of these genes were significantly lower than that in abalone without siRNA injection (*p* < 0.05).

### 3.3. Arginine Treatment of Abalone In Vitro and In Vivo

#### 3.3.1. Expressions of TOR Pathway Related Genes in Hemocyte Treated with Different Concentrations of Arginine

**Figure 7:**
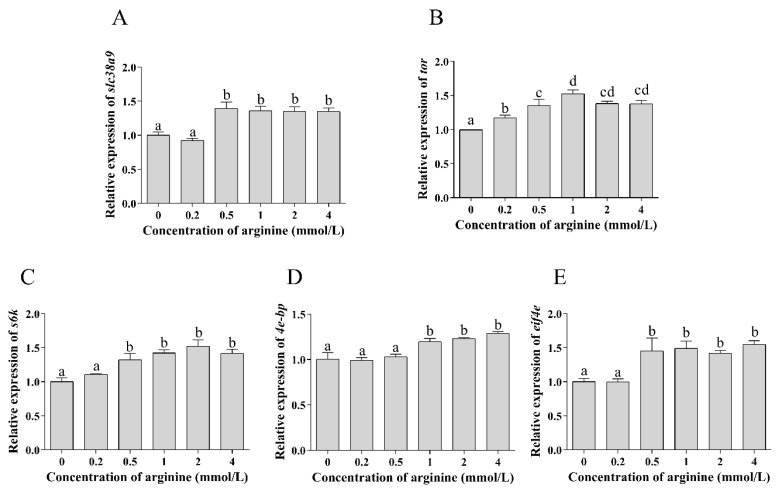
Effect of arginine on TOR signaling pathway of abalone in vitro. The relative expression levels of (**A**) *slc38a9*, (**B**) *tor*, (**C**) *s6k*, (**D**) *4e-bp* and (**E**) *eif4e* of hemocytes treated with different arginine levels (0, 0.2, 0.5, 1, 2 and 4 mmol/L). Results are represented as mean ± SEM (*n* = 3 replicate experiments), and different lowercase letters (a < b < c < d) indicate significant differences among groups (*p* < 0.05).

The relative expression levels of *slc38a9*, *s6k* and *eif4e* were increased significantly when the concentration of arginine was 0.5–4 mmol/L (*p* < 0.05), and there was no significant difference among them (*p* > 0.05). Compared with the control group, the addition of arginine up-regulated the relative expression of *tor* (*p* < 0.05). Furthermore, 1–4 mmol/L arginine significantly increased the relative expression of *4e-bp* (*p* < 0.05) (Figure 7).

#### 3.3.2. Expressions of TOR Pathway Related Genes Affected by Dietary Arginine

**Figure 8:**
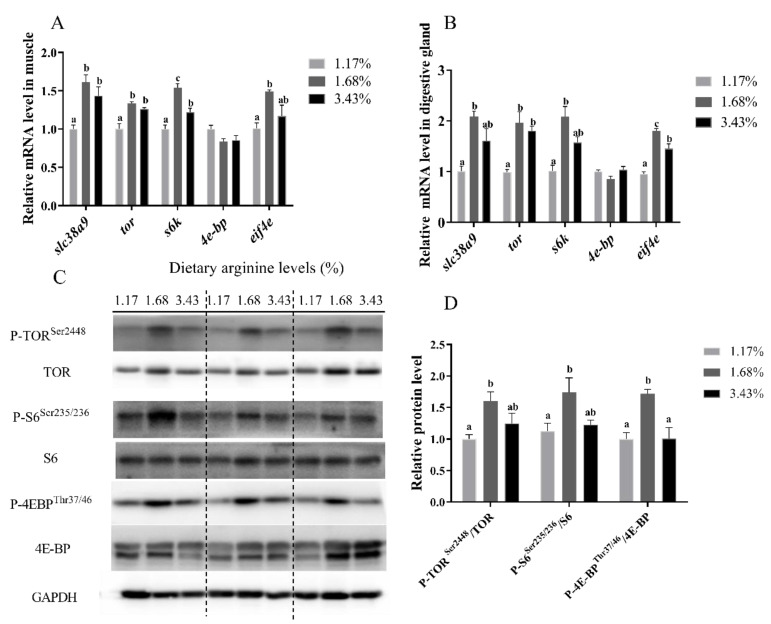
Effect of arginine on TOR signaling pathway of abalone in vivo. The expression levels of *slc38a9*, *tor*, *s6k*, *4e-bp* and *eif4e* in muscle (**A**) and digestive gland (**B**) of abalone fed diets with different arginine levels (1.17%, 1.68% and 3.43%) for 120 d. (**C**,**D**) The abundance of phosphorylated TOR, Ribosomal S6 (S6) and 4E-BP protein in muscle of abalone fed diets with different arginine levels for 120 d. Results are represented as mean ± SEM (*n* = 3 replicate experiments), and different lowercase letters (a < b < c) indicate significant differences among groups (*p* < 0.05).

As presented in Figure 8A, the relative expression levels of *slc38a9* and *tor* in muscle of abalone fed with 1.68% and 3.43% of dietary arginine were significantly higher than those fed diet with 1.17% of arginine (*p* < 0.05). Compared with 1.17% and 3.43% of dietary arginine, diet with 1.68% of arginine significantly increased the expression of *s6k* in abalone (*p* < 0.05). The relative expression levels of *eif4e* in muscle of abalone fed with 1.68% of dietary arginine were significantly higher than those fed with 1.17% of dietary arginine (*p* < 0.05). In addition, there was no significant difference in the mRNA level of *4e-bp* in abalone fed with different levels of dietary arginine (*p* > 0.05).

As for the digestive gland, the relative expression levels of *tor* and *eif4e* in abalone fed with 1.68% and 3.43% of dietary arginine were significantly higher than those fed with diet with 1.17% of arginine (*p* < 0.05). Compared with 1.17% of dietary arginine, 1.68% of dietary arginine significantly improved mRNA levels of *slc38a9* and *s6k* in digestive gland (*p* < 0.05). Dietary arginine level had no significant effect on the expression of *4e-bp* in the digestive gland (*p* > 0.05) (Figure 8B).

#### 3.3.3. Expressions of TOR Pathway Related Proteins Affected by Dietary Arginine

The phosphorylation levels of TOR (Ser^2448^) in muscle of abalone fed with 1.68% of dietary arginine were significantly increased compared to those in abalone fed with 1.17% of dietary arginine (*p* < 0.05). Abalone fed 1.68% of dietary arginine showed the highest phosphorylation level of S6 (Ser^235/236^). Meanwhile, 1.68% of dietary arginine significantly increased the phosphorylation levels of 4E-BP1 (Thr^37/46^) compared with the other two groups (*p* < 0.05) (Figure 8C,D).

## 4. Discussion

SLC38A9 is characterized as a lysosomal membrane amino acid transporter with 11 transmembrane domains and a member of the solute carrier family 38 in human, which has the dual role of transporting amino acids and interacting with other proteins to activate TOR [28,30,36]. The present results showed that the *slc38a9* contains an open reading frame of 1578bp and codes a polypeptide of 525 amino acids with a molecular weight of 58.8 kDa. The sequence analysis in the present study confirmed that SLC38A9 protein has 11 transmembrane domains and no signal peptide, which is in agreement with the results in human [28]. The multiple alignment and phylogenetic tree analysis showed that the *slc38a9* sequence of abalone shares a higher degree of similarity with mollusks such as oyster and scallop, and all mollusk species formed a clade separate from other species, which indicated that *slc38a9* of abalone has high homology with mollusk. In addition, the present study showed that *slc38a9* was expressed in all the analyzed tissues, which is consistent with the expression pattern of the SLC38 family in human and closely related mammals [37]. Transporters of the SLC38 family are particularly expressed in cells with active growth and amino acid metabolism, and SLC38A9 is especially expressed in testis, adrenal gland, thyroid and parathyroid [37,38]. Similarly, this study found that *slc38a9* had the highest expression in the gonad of abalone. Moreover, the transport of arginine is one of the important functions of *slc38a9*; arginine is involved in the sperm formation process and is a basic component of sperm nuclear protein, and it can also improve sperm motility and protect the integrity of sperm structure and function [39,40]. As a metabolite of arginine, NO is essential for sustaining oocyte quality for optimal fertilization and development [41]. The high expression of *slc38a9* may suggest the active transport and utilization of arginine in gonads. Furthermore, the intestine is the main organ of amino acid absorption [42,43], and it may be the reason why *slc38a9* is highly expressed in the intestine in abalone.

Current research generally believes that amino acids regulate the TOR signaling pathway through the Rag-GTPases complex [26,44]. SLC38A9 was identified as a novel component of the Rag-Ragulator machinery and plays an important role in the regulation of the TOR signaling pathway in human cells [28,30]. In HEK-293T cells, the depletion of SLC38A9 decreased the phosphorylation of S6K and 4EBP1, and the phosphorylation of S6K was increased when SLC38A9 was overexpressed [28]. In the present study, the *slc38a9* was artificially regulated by siRNA knockdown or overexpression to confirm the function and the underlying mechanism of SLC38A9 in the regulation of TOR by arginine in abalone. The results showed that knockdown of *slc38a9* inhibited the expression of *tor*, *s6k* and *eif4e*, and the overexpression of *slc38a9* activated the TOR signaling pathway, which is manifested as the increased relative expression level of *tor*, *s6k* and *eif4e*. These results are consistent with studies in human [28,30], suggesting that SLC38A9 plays a positive role in the regulation of the TOR signaling pathway of abalone. Furthermore, studies in human and mouse cells indicated that SLC38A9 is required for the amino acid-dependent TOR activation, and arginine is one of the main activators of this process [30,36]. In HEK-293T cells, siRNA-mediated knockdown of SLC38A9 inhibited the amino acid-induced mTORC1 activation, and knocked out of SLC38A9 suppressed the activation of mTOR by arginine at all concentrations [29,30]. In human HEK293E, HeLa, LN229 cells and mouse embryonic fibroblasts, overexpression of SLC38A9 could activate mTORC1 and sustain its activity during amino acid starvation [28,30]. Consistent with these studies, the present results showed that knockdown of *slc38a9* did partially inhibit the activation of the TOR signaling pathway by arginine, as detected by the mRNA level of *tor* and its established substrate *s6k* and *eif4e*. Therefore, SLC38A9 is essential in the activation of the TOR signaling pathway by arginine.

The TOR signaling pathway plays a crucial role in maintaining cellular and physiological homeostasis [45,46]. Indispensable amino acids have remarkable effects on regulating the TOR pathway, including the regulation of S6K and 4E-BP to promote protein synthesis and initiate translation [47,48,49], and arginine is essential for the activation of TOR [50]. In the present study, 0.5–4 mmol/L arginine significantly increased the expressions of *tor*, *s6k* and *eif4e* in the hemocytes of abalone. It is consistent with the result in bovine mammary epithelial cells [19]. In addition, the present results demonstrated that 1.68% of dietary arginine could activate the TOR signaling pathway in muscle and digestive gland, which is evidenced by the increased relative expression level of *tor*, *s6k* and *eif4e*. Similarly, previous studies in fish species have shown that optimal dietary arginine increased the expression of *tor* and *s6k* [11,21,22,23,24]. Furthermore, dietary arginine levels had no significant effect on the mRNA level of *4e-bp*, which is consistent with studies in tiger puffer, hybrid grouper, blunt snout bream and gibel carp [11,22,23,25], but different results have also been reported in juvenile Jian carp [24], as it suggested that the regulation of arginine on 4E-BP in aquatic animals is complex and requires further relevant study. In the present study, the phosphorylation levels of TOR, 4E-BP and S6 were detected for a comprehensive understanding of the regulation of arginine on the TOR signaling pathway. As 4E-BP is the binding protein of eIF4E, phosphorylation of 4E-BP releases eIF4E and initiates translation [51], and dietary arginine supplementation promoted the formation of the eIF4E·eIF4G complex and reduced the amount of the 4E-BP1·eIF4E complex in skeletal muscle of piglets [20]. Arginine increased the abundance of phosphorylated mTOR, S6K and 4E-BP1 proteins in porcine and ovine trophectoderm cells and brown adipocyte precursor cells [16,17,18]. S6 is the primary substrate of S6K, and it is directly regulated by S6K to regulate protein synthesis [52], the abundance of phosphorylated S6 and S6K was increased by arginine in porcine trophectoderm cells [53]. The present study showed that 1.68% dietary arginine increased phosphorylation levels of TOR, S6 and 4E-BP in muscle. It showed a similar trend in mammals [17,18,20], suggesting that 1.68% dietary arginine activated the TOR signaling pathway at the protein phosphorylation level. Furthermore, excessive metabolism of amino acids may result in excessive energy cost and toxicity, 3.43% dietary arginine may exceed the tolerance level of arginine in abalone, resulting in an imbalance of amino acid, thereby inhibiting the activity of related genes and metabolic processes. Taken together, appropriate levels of arginine activated the TOR signaling pathway, which is evidenced by the increased expression levels of *tor, s6k* and *eif4e* in hemocytes, muscle and digestive gland. A total of 1.68% of dietary arginine increased the abundance of phosphorylated TOR, 4E-BP and S6 in muscle. These results suggested a promotion of protein synthesis in abalone.

The gene transcription of several members from the SLC superfamily are transcriptionally affected by amino acid levels [51,54]. Ingestion of essential amino acids increased the mRNA levels of SLC38A9 in human [55]. In piglets, the relative expression of neutral amino acid transporter *slc1a5* was increased by L-glutamate, and leucine increased the expression of *slc6a14, slc6a19* and *slc7a9* [56,57]. An optimal level of dietary arginine up-regulated the expressions of cationic amino acid transporters in spotted grouper (*Epinephelus coioides*) [58] and tiger puffer (*Takifugu rubripes)* [25]. Similarly, the present study showed that 1.68% and 3.43% of dietary arginine increased the relative expression levels of *slc38a9* in muscles and digestive gland of abalone (*p* < 0.05). The result indicated that appropriate arginine could increase the activation of amino acid transporter, which can promote the transport and utilization of arginine. In addition, the expression of *slc38a9* is consistent with the expression trend of TOR-related genes, which supported the regulation of the TOR signaling pathway by SLC38A9.

The TOR signaling pathway has also emerged as a key regulator of autophagy and inflammatory response [49], and deregulation of TOR underlies the pathogenesis of metabolic disorders, cancer, neurodegeneration and aging [59,60]. SLC38A9 mediates the transport of essential amino acids out of lysosomes, which is required for the growth of pancreatic cancer cells, and loss of SLC38A9 or its transport function strongly inhibited tumor formation [61]. Furthermore, pancreatic cancers have more than 90% KRAS mutation and KRAS is one of the most frequently mutated oncogenes of the RAS family, and it is reported that combinatorial therapies with TOR inhibitors will be necessary against RAS-driven cancers [62]. An antagonist of SLC38A9 (NOVELTY) was developed for the treatment of proliferative disease and metabolic disorder associated with TOR activation [63]. The present study in abalone demonstrated that SLC38A9 is conservative in regulating the TOR signaling pathway and provided further evidence that SLC38A9 may be used as a promising anticancer target against related cancers. Moreover, amino acids and TOR were reported to mediate nutritional checkpoints in the cell cycle, which were usually inactivated and/or overridden in cancer cells [64,65]. Arginine depletion is considered as a viable option for cancer treatment, it results in the induction of cell growth arrest at several checkpoints [66,67], and the present study also showed that arginine deficiency inhibited the TOR signaling pathway. Therefore, exploring the role of arginine and the regulation of TOR might have some potential for therapeutic exploitation.

## 5. Conclusions

**Figure 9:**
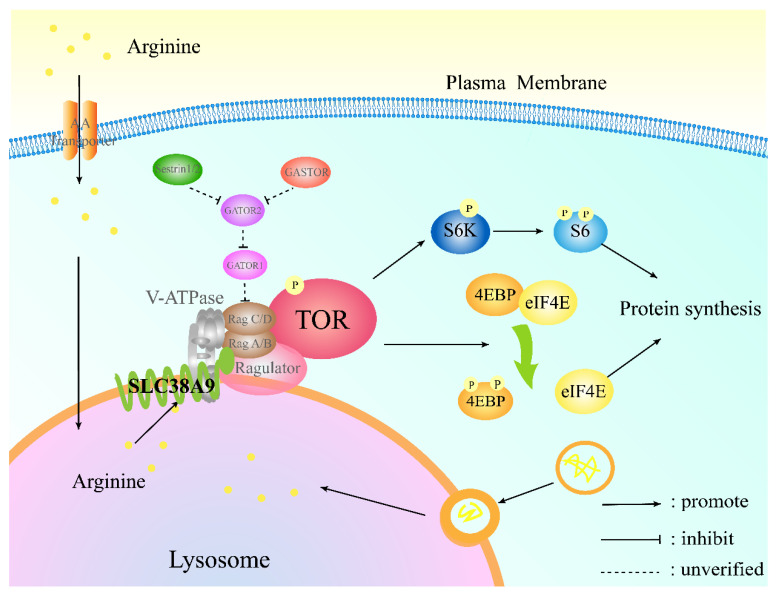
The potential mechanism of arginine on the TOR signaling pathway of abalone *H. discus hannai* in the present study. Arginine activates TOR through SLC38A9, and then the phosphorylation of S6 and 4EBP and the expression of *s6k* and *eif4e* were activated to promote protein synthesis. The unidentified proteins in abalone are shown in grey.

In conclusion, the sequence of *slc38a9* in abalone was cloned. It shows high sequence homology to other mollusks and is widely expressed in various tissues. SLC38A9 plays a positive role in the regulation of the TOR signaling pathway, and it is essential in the activation of TOR by arginine. Moreover, appropriate levels of arginine increased the activity of the TOR signaling pathway and *slc38a9* in vivo and in vitro (Figure 9). This study provides insights into the potential mechanism of the regulation of the TOR signaling pathway by arginine and the molecular basis for the promotion of dietary amino acids on protein synthesis and growth.

## Figures and Tables

**Table 1:** Sequences of primers for gene cloning and RT-PCR.

Name	Primer Sequence (5′ to 3′)	Accession No.
Gene Cloning		
*slc38a9*	F: GAGGCGGCTATGGGTCAAT R: CTGAGAACAGGTCCGAGGT	MW390888
Real-Time PCR		
*tor*	F: AGATTCCCTTCCGATTGACGA R: GTACGGCCATCAGACTGTCC	MT473702
*s6k*	F: GCCCCTGCTTTACTCGATG R: CAGCTCTTCACACCCGGTA	MT497737
*4e-bp*	F: ATCGGTCTTTCTTACTGGAATGTCG R: AGGCTGTTCTTCAGGGTGGTC	MT497738
*eif4e*	F: AGAATCAGCGTTGTATCACCT R: TGCGAGAATCTTCCCATGCC	MW183129
*slc38a9*	F: CGCCATGTCCTGATGCTC R: TGGCATACGAGAACCCACA	MW390888
*β-actin*	F: ACTCCATCATGAAGTGCGAT R: TTCTGCATACGGTCAGCGAT	AY380809.1

**Table 2:** Sequences of siRNA-*slc38a9* for different sites.

Site	Forward (5′-3′)	Reverse (5′-3′)
siRNA-108	GCTACTCAGTCGCTACAAA	TTTGTAGCGACTGAGTAGC
siRNA-236	GCAGCATCATCACCATCTT	AAGATGGTGATGATGCTGC
siRNA-347	CCAGCTACAGAATACTCAA	TTGAGTATTCTGTAGCTGG
siRNA-469	GCAGAAATAGGAGCAGTTA	TAACTGCTCCTATTTCTGC

**Table 3:** Ingredients and proximate analysis of the experimental diets.

Ingredients	Dietary Arginine Levels (%)	
	1.23	1.72
Casein	22	22
Gelatin	5	5
Dextrin	35	35
Fish oil + Soybean oil ^a^	3.5	3.5
CM-cellulose	6	6
Microcrystalline cellulose	1.5	1
Sodium alginate	20	20
Mineral mix ^b^	4.5	4.5
Vitamin mix ^c^	2	2
Choline chloride	0.5	0.5
Arginine	0	0.5
**Proximate analysis (% dry matter)**		
Moisture	28.8	28.4
Crude protein	28.7	29.4
Crude lipid	3.84	3.67
Arginine	1.23	1.72

^a^ Fish oil: soybean oil = 1:1. ^b^ Mineral premix (each/kg diet): NaCl, 0.4 g; MgSO_4_·7H_2_O, 6.0 g; NaH_2_PO_4_·2H_2_O, 10.0 g; KH_2_PO_4_, 12.8 g; Ca (H_2_PO_4_)_2_·H_2_O, 8.0 g; Fe-citrate, 1.0 g; calcium lactate, 1.4 g; ZnSO_4_·7H_2_O, 141 mg; MnSO_4_·H_2_O, 64.8 mg; CuSO_4_·5H_2_O, 12.4 mg; CoCl_2_·6H_2_O, 4 mg; KIO_3_, 1.2 mg; microcrystalline cellulose, 4.85 g. ^c^ Vitamin premix (each/kg diet): thiamine, 120 mg; riboflavin, 100 mg; folic acid, 30 mg; nicotinic acid, 800 mg; vitamin B6, 40 mg; calcium pantothenate, 200 mg; inositol, 4 g; biotin, 12.0 mg; vitamin C, 4 g; vitamin B12, 0.18 mg; vitamin A, 1.0 × 10^5^ IU; vitamin D, 2.0 × 10^3^ IU; vitamin E, 450 IU; vitamin K3, 80 mg; microcrystalline cellulose, 1.26 g.

**Table 4:** Ingredients and proximate analysis of the experimental diets.

Ingredients	Dietary Arginine Levels (%)		
	1.17	1.68	3.43
Fish meal	3	3	3
Soy protein concentrate	3	3	3
Corn gluten meal	24	24	24
Wheat gluten	5	5	5
High gluten flour	25	25.2	25.2
Fish oil + Soybean oil ^a^	1.6	1.6	1.6
Calcium dihydrogen phosphate	2	2	2
Choline chloride	0.2	0.2	0.2
Mineral mixture ^b^	1	1	1
Vitamin mixture ^c^	1	1	1
Ethoxyquinoline	0.1	0.1	0.1
Calcium propionate	0.1	0.1	0.1
Vitamin C	0.4	0.4	0.4
Kelp powder	31	31	31
L-arginine	0	0.8	2.4
L-glycine	2.4	1.6	0
**Proximate Analysis (% dry matter)**			
Moisture	4.44	5.32	4.52
Crude protein	32.2	31.9	32.5
Crude lipid	2.98	2.95	3.02
Ash	17.6	16.8	17.6
Arginine	1.17	1.68	3.43

^a^ Fish oil: soybean oil = 1:1. ^b^ Mineral premix (each/100g premix): NaCl, 1 g; MgSO_4_·7H_2_O, 15 g; NaH_2_PO_4_·2H_2_O, 25 g; KH_2_PO_4_, 32 g; Ca (H_2_PO_4_)_2_·H_2_O, 20 g; Fe-citrate, 2.5 g; calcium lactate, 3.5 g; ZnSO_4_·7H_2_O, 0.35 g; MnSO_4_·H_2_O, 0.16 g; CuSO_4_·5H_2_O, 31 mg; CoCl_2_·6H_2_O, 1 mg; KIO_3_, 3 mg. ^c^ itamin premix (each/100g premix): thiamine, 1.2 g; riboflavin, 1 g; folic acid, 300 mg; nicotinic acid, 8 g; vitamin B6, 400 mg; calcium pantothenate, 2 g; inositol, 40 g; biotin, 120 mg; vitamin B12, 1.8 mg; vitamin A, 1.0 × 10^6^ IU; vitamin D, 2 × 10^4^ IU; vitamin E, 450 IU; vitamin K3, 800 mg; microcrystalline cellulose, 2.69 g.

## Data Availability

The data presented in this study are available on request from the corresponding author.

## References

[1] Wu G., Bazer F.W., Davis T.A., Kim S.W., Li P., Marc R.J., Carey S.M., Smith S.B., Spencer T.E., Yin Y. (2009). Arginine metabolism and nutrition in growth, health and disease. Amino. Acids..

[2] Li P., Mai K., Trushenski J., Wu G. (2009). New developments in fish amino acid nutrition: Towards functional and environmentally oriented aquafeeds. Amino. Acids..

[3] Wu G. (2010). Functional amino acids in growth, reproduction, and health. Adv. Nutr..

[4] Jobgen W.S., Fried S.K., Fu W.J., Meininger C.J., Wu G. (2006). Regulatory role for the arginine-nitric oxide pathway in metabolism of energy substrates. J. Nutr. Biochem..

[5] Pohlenz C., Buentello A., Miller T., Small B.C., MacKenzie D.S., Gatlin D.M. (2013). Effects of dietary arginine on endocrine growth factors of channel catfish, Ictalurus punctatus. Comp. Biochem. Physiol. A Mol Integr. Physiol..

[6] Wang L., Wu J., Wang C., Li J., Zhao Z., Luo L., Du X., Xu Q. (2017). Dietary arginine requirement of juvenile hybrid sturgeon (*Acipenser schrenckii*♀ × *Acipenser baerii*♂). Aquac. Res..

[7] Andoh T. (2014). Stress inhibits insulin release induced by feeding and arginine injection in barfin flounder *Verasper moseri*. Fish. Sci..

[8] Sink T.D., Lochmann R.T. (2007). Insulin Response of Largemouth Bass to Glucose, Amino Acid, and Diet Stimulation. N. Am. J. Aquac..

[9] Chen Q., Zhao H., Huang Y., Cao J., Wang G., Sun Y., Li Y. (2016). Effects of dietary arginine levels on growth performance, body composition, serum biochemical indices and resistance ability against ammonia-nitrogen stress in juvenile yellow catfish (*Pelteobagrus fulvidraco*). Anim. Nutr..

[10] Rønnestad I., Thorsen A., Finn R.N. (1999). Fish larval nutrition: A review of recent advances in the roles of amino acids. Aquaculture.

[11] Liang H., Ren M., Habte-Tsion H.M., Ge X., Xie J., Mi H., Xi B., Miao L., Liu B., Zhou Q. (2016). Dietary arginine affects growth performance, plasma amino acid contents and gene expressions of the TOR signaling pathway in juvenile blunt snout bream, *Megalobrama amblycephala*. Aquaculture.

[12] Scot R.K., Leonard S.J. (2002). Control of protein synthesis by amino acid availability. Curr. Opin. Clin. Nutr. Metab. Care.

[13] Wullschleger S., Loewith R., Hall M.N. (2006). TOR signaling in growth and metabolism. Cell.

[14] Kilberg M.S., Pan Y.X., Chen H., Leung-Pineda V. (2005). Nutritional control of gene expression: How mammalian cells respond to amino acid limitation. Annu. Rev. Nutr..

[15] Chotechuang N., Azzout-Marniche D., Bos C., Chaumontet C., Gausseres N., Steiler T., Gaudichon C., Tome D. (2009). mTOR, AMPK, and GCN2 coordinate the adaptation of hepatic energy metabolic pathways in response to protein intake in the rat. Am. J. Physiol. Endocrinol. Metab..

[16] Kim J.Y., Burghardt R.C., Wu G., Johnson G.A., Spencer T.E., Bazer F.W. (2011). Select nutrients in the ovine uterine lumen. VIII. Arginine stimulates proliferation of ovine trophectoderm cells through MTOR-RPS6K-RPS6 signaling cascade and synthesis of nitric oxide and polyamines. Biol. Reprod..

[17] Kong X., Tan B., Yin Y., Gao H., Li X., Jaeger L.A., Bazer F.W., Wu G. (2012). L-Arginine stimulates the mTOR signaling pathway and protein synthesis in porcine trophectoderm cells. J. Nutr. Biochem..

[18] Ma X., Han M., Li D., Hu S., Gilbreath K.R., Bazer F.W., Wu G. (2017). L-Arginine promotes protein synthesis and cell growth in brown adipocyte precursor cells via the mTOR signal pathway. Amino. Acids..

[19] Wang M., Xu B., Wang H., Bu D., Wang J., Loor J.J. (2014). Effects of Arginine concentration on the in vitro expression of Casein and mTOR pathway related genes in mammary epithelial cells from dairy cattle. PLoS ONE.

[20] Yao K., Yin Y., Chu W., Liu Z., Deng D., Li T., Huang R., Zhang J., Tan B., Wang W. (2008). Dietary Arginine Supplementation Increases mTOR Signaling Activity in Skeletal Muscle of Neonatal Pigs. J. Nutr..

[21] Chen J., Zhang D., Tan Q., Liu M., Hu P. (2019). Arginine affects growth and integrity of grass carp enterocytes by regulating TOR signaling pathway and tight junction proteins. Fish Physiol. Biochem..

[22] Wu M., Wu X., Lu S., Gao Y., Yao W., Li X., Dong Y., Jin Z. (2018). Dietary arginine affects growth, gut morphology, oxidation resistance and immunity of hybrid grouper (*Epinephelus fuscoguttatus* ♀ × *Epinephelus lanceolatus* ♂) juveniles. Br. J. Nutr..

[23] Tu Y., Xie S., Han D., Yang Y., Jin J., Zhu X. (2015). Dietary arginine requirement for gibel carp (*Carassis auratus gibelio* var. CAS ⅠⅠⅠ ) reduces with fish size from 50g to 150g associated with modulation of genes involved in TOR signaling pathway. Aquaculture.

[24] Chen G., Feng L., Kuang S., Liu Y., Jiang J., Hu K., Jiang W., Li S., Tang L., Zhou X. (2012). Effect of dietary arginine on growth, intestinal enzyme activities and gene expression in muscle, hepatopancreas and intestine of juvenile Jian carp (*Cyprinus carpio* var. Jian). Br. J. Nutr..

[25] Wei Y., Zhang Q., Jia L., Xu H., Liang M. (2021). Effects of dietary arginine levels on growth, intestinal peptide and amino acid transporters, and gene expressions of the TOR signaling pathway in tiger puffer, Takifugu rubripes. Aquaculture.

[26] Sancak Y., Peterson T.R., Shaul Y.D., Lindquist R.A., Thoreen C.C., Bar-Peled L., Sabatini D.M. (2008). The Rag GTPases bind raptor and mediate amino acid signaling to mTORC1. Science.

[27] Bar-Peled L., Schweitzer L.D., Zoncu R., Sabatini D.M. (2012). Ragulator is a GEF for the rag GTPases that signal amino acid levels to mTORC1. Cell.

[28] Jung J., Genau H.M., Behrends C. (2015). Amino Acid-Dependent mTORC1 Regulation by the Lysosomal Membrane Protein SLC38A9. Mol. Cell Biol..

[29] Rebsamen M., Pochini L., Stasyk T., de Araujo M.E., Galluccio M., Kandasamy R.K., Snijder B., Fauster A., Rudashevskaya E.L., Bruckner M. (2015). SLC38A9 is a component of the lysosomal amino acid sensing machinery that controls mTORC1. Nature.

[30] Wang S., Tsun Z.Y., Wolfson R.L., Shen K., Wyant G.A., Plovanich M.E., Yuan E.D., Jones T.D., Chantranupong L., Comb W. (2015). Metabolism. Lysosomal amino acid transporter SLC38A9 signals arginine sufficiency to mTORC1. Science.

[31] Wu C., Zhang W., Mai K., Xu W., Wang X., Ma H., Liufu Z. (2010). Transcriptional up-regulation of a novel ferritin homolog in abalone *Haliotis discus hannai* Ino by dietary iron. Comp. Biochem. Physiol. C Toxicol. Pharm..

[32] Ma S., Guo Y., Sun L., Fan W., Liu Y., Liu D., Huang D., Li X., Zhang W., Mai K. (2020). Over high or low dietary protein levels depressed the growth, TOR signaling, apoptosis, immune and anti-stress of abalone *Haliotis discus hannai*. Fish Shellfish Immunol..

[33] Guo Y., Huang D., Chen F., Ma S., Zhou W., Zhang W., Mai K. (2021). Lipid deposition in abalone *Haliotis discus hannai* affected by dietary lipid levels through AMPKα2/PPARα and JNK/mTOR/SREBP-1c pathway. Aquaculture.

[34] Wang L., Guo Y., Pan M., Li X., Huang D., Liu Y., Wu C., Zhang W., Mai K. (2021). Functions of Forkhead Box O on Glucose Metabolism in Abalone *Haliotis discus hannai* and Its Responses to High Levels of Dietary Lipid. Genes.

[35] Liu J., Pan M., Huang D., Guo Y., Yang M., Zhang W., Mai K. (2020). Myostatin-1 Inhibits Cell Proliferation by Inhibiting the mTOR Signal Pathway and MRFs, and Activating the Ubiquitin-Proteasomal System in Skeletal Muscle Cells of Japanese Flounder Paralichthys olivaceus. Cells.

[36] Scalise M., Galluccio M., Pochini L., Cosco J., Trotta M., Rebsamen M., Superti-Furga G., Indiveri C. (2019). Insights into the transport side of the human SLC38A9 transceptor. Biochim Biophys Acta. Biomembr..

[37] Broer S. (2014). The SLC38 family of sodium-amino acid co-transporters. Pflug. Arch..

[38] Hellsten S.V., Eriksson M.M., Lekholm E., Arapi V., Perland E., Fredriksson R. (2017). The gene expression of the neuronal protein, SLC38A9, changes in mouse brain after in vivo starvation and high-fat diet. PLoS ONE.

[39] Patel A.B., Srivastava S., Phadke R.S., Govil G. (1998). Arginine Activates Glycolysis of Goat Epididymal Spermatozoa: An NMR Study. Biophys. J..

[40] (1970). Adnan; Mroueh, Effect of Arginine on Oligospermia. Fertil. Steril..

[41] Goud P.T., Goud A.P., Diamond M.P., Gonik B., Abu-Soud H.M. (2008). Nitric oxide extends the oocyte temporal window for optimal fertilization. Free Radic. Biol. Med..

[42] Stein E.D., Chang S.D., Diamond J.M. (1987). Comparison of different dietary amino acids as inducers of intestinal amino acid transport. Am. J. Physiol..

[43] Morales A., Barrera M.A., Araiza A.B., Zijlstra R.T., Bernal H., Cervantes M. (2013). Effect of excess levels of lysine and leucine in wheat-based, amino acid-fortified diets on the mRNA expression of two selected cationic amino acid transporters in pigs. J Anim. Physiol. Anim. Nutr..

[44] Kim E., Goraksha-Hicks P., Li L., Neufeld T.P., Guan K.L. (2008). Regulation of TORC1 by Rag GTPases in nutrient response. Nat. Cell Biol..

[45] Holz M.K., Ballif B.A., Gygi S.P., Blenis J. (2005). mTOR and S6K1 mediate assembly of the translation preinitiation complex through dynamic protein interchange and ordered phosphorylation events. Cell.

[46] Laplante M., Sabatini D.M. (2012). mTOR signaling in growth control and disease. Cell.

[47] Sancak Y., Bar-Peled L., Zoncu R., Markhard A.L., Nada S., Sabatini D.M. (2010). Ragulator-Rag complex targets mTORC1 to the lysosomal surface and is necessary for its activation by amino acids. Cell.

[48] Hara K., Yonezawa K., Weng Q.P., Kozlowski M.T., Avruch J. (1998). Amino acid Sufficiency and mTOR Regulate p70 S6 Kinase and eIF-4E BP1 through a Common Effector Mechanism. J. Biol. Chem..

[49] Habte-Tsion H.M. (2020). A review on fish immuno-nutritional response to indispensable amino acids in relation to TOR, NF-kappaB and Nrf2 signaling pathways: Trends and prospects. Comp. Biochem. Physiol. B Biochem. Mol. Biol..

[50] Carroll B., Maetzel D., Maddocks O.D., Otten G., Ratcliff M., Smith G.R., Dunlop E.A., Passos J.F., Davies O.R., Jaenisch R. (2016). Control of TSC2-Rheb signaling axis by arginine regulates mTORC1 activity. Elife.

[51] Taylor P.M. (2014). Role of amino acid transporters in amino acid sensing. Am. J. Clin. Nutr..

[52] Ruvinsky I., Meyuhas O. (2006). Ribosomal protein S6 phosphorylation: From protein synthesis to cell size. Trends Biochem. Sci..

[53] Kim J., Song G., Wu G., Gao H., Johnson G.A., Bazer F.W. (2013). Arginine, leucine, and glutamine stimulate proliferation of porcine trophectoderm cells through the MTOR-RPS6K-RPS6-EIF4EBP1 signal transduction pathway. Biol. Reprod..

[54] Hellsten S.V., Tripathi R., Ceder M.M., Fredriksson R. (2018). Nutritional Stress Induced by Amino Acid Starvation Results in Changes for Slc38 Transporters in Immortalized Hypothalamic Neuronal Cells and Primary Cortex Cells. Front Mol. Biosci..

[55] Graber T.G., Borack M.S., Reidy P.T., Volpi E., Rasmussen B.B. (2017). Essential amino acid ingestion alters expression of genes associated with amino acid sensing, transport, and mTORC1 regulation in human skeletal muscle. Nutr. Metab..

[56] Sun Y., Wu Z., Li W., Zhang C., Sun K., Ji Y., Wang B., Jiao N., He B., Wang W. (2015). Dietary L-leucine supplementation enhances intestinal development in suckling piglets. Amino. Acids..

[57] Lin M., Zhang B., Yu C., Li J., Zhang L., Sun H., Gao F., Zhou G. (2014). L-Glutamate supplementation improves small intestinal architecture and enhances the expressions of jejunal mucosa amino acid receptors and transporters in weaning piglets. PLoS ONE.

[58] Cui X., Han F., Chi S., Tan B., Dong X., Yang Q., Liu H., Zhang S. (2020). Molecular cloning of the amino acid transporter b0,+AT cDNA from the orange-spotted grouper (*Epinephelus coioides*) and the effect of arginine on its expression. Aquac. Nutr..

[59] Liu G.Y., Sabatini D.M. (2020). mTOR at the nexus of nutrition, growth, ageing and disease. Nat. Rev. Mol. Cell Biol..

[60] Huang S. (2020). mTOR Signaling in Metabolism and Cancer. Cells.

[61] Wyant G.A., Abu-Remaileh M., Wolfson R.L., Chen W.W., Freinkman E., Danai L.V., Vander Heiden M.G., Sabatini D.M. (2017). mTORC1 Activator SLC38A9 Is Required to Efflux Essential Amino Acids from Lysosomes and Use Protein as a Nutrient. Cell.

[62] Mukhopadhyay S., Vander Heiden M.G., McCormick F. (2021). The Metabolic Landscape of RAS-Driven Cancers from biology to therapy. Nat. Cancer.

[63] Rebsamen M., Superti-Furga G. (2015). Antagonists of slc38a9 and their use in therapy.

[64] Saqcena M., Menon D., Patel D., Mukhopadhyay S., Chow V., Foster D.A. (2013). Amino acids and mTOR mediate distinct metabolic checkpoints in mammalian G1 cell cycle. PLoS ONE.

[65] Mukhopadhyay S., Saqcena M., Foster D.A. (2015). Synthetic lethality in KRas-driven cancer cells created by glutamine deprivation. Oncoscience.

[66] Kumari N., Bansal S. (2021). Arginine depriving enzymes: Applications as emerging therapeutics in cancer treatment. Cancer Chemother Pharm..

[67] Du T., Han J. (2021). Arginine Metabolism and Its Potential in Treatment of Colorectal Cancer. Front. Cell Dev. Biol..

